# The Development and Characterization of a Human Mesothelioma *In Vitro* 3D Model to Investigate Immunotoxin Therapy

**DOI:** 10.1371/journal.pone.0014640

**Published:** 2011-01-31

**Authors:** Xinran Xiang, Yen Phung, Mingqian Feng, Kunio Nagashima, Jingli Zhang, V. Courtney Broaddus, Raffit Hassan, David FitzGerald, Mitchell Ho

**Affiliations:** 1 Laboratory of Molecular Biology, Center for Cancer Research, National Cancer Institute, National Institutes of Health, Bethesda, Maryland, United States of America; 2 Science Applications International Corporation-Frederick, Inc., Frederick, Maryland, United States of America; 3 Lung Biology Center, University of California San Francisco, San Francisco, California, United States of America; Tufts University, United States of America

## Abstract

**Background:**

Tumor microenvironments present significant barriers to penetration by antibodies and immunoconjugates. Tumor microenvironments, however, are difficult to study *in vitro*. Cells cultured as monolayers exhibit less resistance to therapy than those grown *in vivo* and an alternative research model more representative of the *in vivo* tumor is more desirable. SS1P is an immunotoxin composed of the Fv portion of a mesothelin-specific antibody fused to a bacterial toxin that is presently undergoing clinical trials in mesothelioma.

**Methodology/Principal Findings:**

Here, we examined how the tumor microenvironment affects the penetration and killing activity of SS1P in a new three-dimensional (3D) spheroid model cultured *in vitro* using the human mesothelioma cell line (NCI-H226) and two primary cell lines isolated from the ascites of malignant mesothelioma patients. Mesothelioma cells grown as monolayers or as spheroids expressed comparable levels of mesothelin; however, spheroids were at least 100 times less affected by SS1P. To understand this disparity in cytotoxicity, we made fluorescence-labeled SS1P molecules and used confocal microscopy to examine the time course of SS1P penetration within spheroids. The penetration was limited after 4 hours. Interestingly, we found a significant increase in the number of tight junctions in the core area of spheroids by electron microscopy. Expression of E-Cadherin, a protein involved in the assembly and sealing of tight junctions and highly expressed in malignant mesothelioma, was found significantly increased in spheroids as compared to monolayers. Moreover, we found that siRNA silencing and antibody inhibition targeting E-Cadherin could enhance SS1P immunotoxin therapy *in vitro*.

**Conclusion/Significance:**

This work is one of the first to investigate immunotoxins in 3D tumor spheroids *in vitro*. This initial description of an *in vitro* tumor model may offer a simple and more representative model of *in vivo* tumors and will allow for further investigations of the microenvironmental effects on drug penetration and tumor cell killing. We believe that the methods developed here may apply to the studies of other tumor-targeting antibodies and immunoconjugates *in vitro*.

## Introduction

Solid tumors remain a major problem despite a vast number of anti-cancer agents available. Advances in antibody-based therapies represent a promising new approach to treating solid cancer, yet a major challenge involves delivering sufficient amounts of antibodies and immunoconjugates within tumor masses [1]. For an anti-cancer antibody agent to be successful, it must satisfy two requirements: (a) the agent must be effective in the tumor microenvironment, and (b) the agent must reach the tumor cells in optimal quantities.

Tumor microenvironments are difficult to study *in vivo* and have been extensively studied primarily at the monolayer level *in vitro*. Cancer cells cultured as monolayers exhibit less resistance to therapy than those grown *in vivo* and may be explained by “multicellular resistance,” a mechanism for drug resistance attributed to cell-cell contacts, cell-matrix contacts, and the three-dimensional (3D) shape found in tissue [2]–[4]. Multicellular resistance acquired by tumor cells may contribute to difficulties in translating promising findings from *in vitro* studies into therapy [5]. *In vitro* multicellular cancer spheroids, therefore, have begun to bridge the complexity gap between monolayer cell culture and *in vivo* tumors and have become valuable models in the study of drug resistance [6].

Mesothelioma is a fatal cancer of the mesothelium and predominantly forms from previous exposure to asbestos [7]. Malignant mesothelioma (MM) is often resistant to chemotherapy [8] and radiation [9]. Prognosis is poor and average survival ranges from a few months to less than 2 years [10]. To investigate apoptotic resistance in mesothelioma, Broaddus and colleagues recently reported that mesothelioma cells acquired resistance when formed into 3D spheroids *in vitro* [11]. These spheroids exhibited many features of the tumor microenvironment, modeling the avascular region of tumors that is dependent on diffusion. Antibody-based therapy has emerged as a new promising strategy to treat mesothelioma and other solid tumors [12]. However, tumor microenvironments that present significant barriers to penetration by antibodies and immunoconjugates have been studied only *in vivo*. A simple, reliable, high-throughput and less expensive *in vitro* tumor model should be very useful for characterizing and screening antibodies and immunoconjugates for cancer therapy.

Mesothelin is a tumor differentiation antigen that is normally expressed in low levels on the mesothelial cells lining the pleura, peritoneum and pericardium [12]. Mesothelin is highly expressed in mesothelioma, as well as ovarian cancer and lung cancer [12], [13], and has been shown to be a biomarker for the diagnosis of mesothelioma [14]. Although the biological function of mesothelin remains unclear, mesothelin's limited expression in normal tissue and high expression in various cancers make it an attractive candidate for immunotherapy [12]. The mucin CA125/MUC16 is also highly expressed at the cell surface in mesothelioma and ovarian cancer [15]. The binding of mesothelin to CA125/MUC16 may play a role in the implantation and peritoneal spread of tumors by cell adhesion [15]. The recombinant immunotoxin SS1P is currently in clinical trials for mesothelioma. SS1P is composed of the Fv portion of an anti-mesothelin monoclonal antibody (mAb) fused to a 38 kDa *Pseudomonas* exotoxin-A (PE) fragment [12]. After binding to mesothelin, the immunotoxin is internalized, undergoes processing in the endocytic compartment and the immunotoxin fragment containing the ADP-ribosylation domain is transported to the endoplasmic reticulum. It is then translocated to the cytosol where it inhibits elongation factor-2 leading to inhibition of protein synthesis and ultimately cell death.

The goal of the present work is to establish a basic *in vitro* 3D spheroid model of human mesothelioma and to investigate how the tumor microenvironment affects the penetration and killing activity of the immunotoxin SS1P targeting mesothelioma. This approach shows that 3D tumor microenvironments increase the number of tight junctions and inhibit SS1P penetration within tumor spheroids. We also demonstrate how this new method can be used to identify potential new therapeutic targets (e.g., E-Cadherin) highly expressed in 3D mesothelioma, but not in monolayers. The method developed here is simple and may easily apply to the studies of other tumor-targeting antibodies and immunoconjugates *in vitro*.

## Methods

### Cell lines

The human mesothelioma cell line NCI-H226 was obtained from the American Type Culture Collection (ATCC; Rockville, MD). The cell line was maintained as adherent monolayer cultures in RPMI 1640 medium (Invitrogen, Carlsbad, CA) supplemented with 10% fetal bovine serum (HyClone, Logan, UT), L-glutamine, pyruvate, nonessential amino acids, and penicillin-streptomycin (Invitrogen) and incubated in 5% CO_2_ with balance of air at 37°C. Cells were seeded at 2×10^5^/mL in T-75 tissue culture flasks (Corning Incorporated, Acton, MA). After four days of growth, this cell density did not produce confluence. Cells were harvested and the media were changed twice a week. Cells were confirmed to be negative for mycoplasma.

The two primary cell lines (NCI-M-03 and NCI-M-13) were established from the ascites of MM patients being treated at the National Cancer Institute (NCI). The ascites were obtained on Institutional Review Board approved protocols and patients signed informed consent. Briefly, the ascites (100–1000 mL) were collected and spun down at 1000 rpm at room temperature for 5 minutes; the cell pellets were washed twice with PBS, red blood cells were removed by BD Pharm LyseTM-Lysing Buffer kit (BD Bioscience, Franklin Lakes, NJ) according to the manufacturer's instructions. The cells were resuspended in RPMI 1640 (Invitrogen) supplemented with 20% fetal bovine serum (Lonza, Walkersville, MD), 2 mM Glutamine (Invitrogen), 100 units penicillin-streptomycin (Invitrogen), and 1 mM Sodium Pyruvate (Invitrogen). The cells were seeded into 175 mL culture flasks at a density of 2.5–4.0×10^5^ cells/mL. After a 24 hour incubation at 37°C in a humidified, 5% CO_2_ atmosphere overnight, the medium containing non-adherent cells was replaced with fresh medium. The media were changed twice a week.

### Spheroid formation

A 96-well Greiner suspension culture plate (Sigma, St. Louis, MO) was coated with 50 µL of 5 mg/mL of poly-HEMA (poly-2-hydroxyethyl methacrylate; Sigma-Aldrich) in 95% ethanol and evaporated with lid on at room temperature for 72 hours [11]. Mesothelioma cells were grown to near confluency and dissociated into single cells with Accutase (BD Biosciences, San Jose, CA). Each well contained 10,000 cells for one spheroid. The plate was then centrifuged at 1000 rpm for 10 minutes to initiate cell-cell interaction and incubated at 37°C, 5% CO_2_ for 24 hours. The spheroids are stable for 48–72 hours and can be easily transferred using a regular pipette without dissociating.

To treat spheroids with an inhibitory mAb against E-Cadherin, a pretreatment group of NCI-H226 cells was incubated with 1 µg/mL of SHE78-7 (cat. #13-5700; Invitrogen) for 30 minutes and then seeded as spheroids. The post-treatment group of cells was seeded as spheroids and then incubated with 1 µg/mL of SHE78-7 for 30 minutes. Both groups of spheroids were incubated for 48 hours and then treated with different concentrations of SS1P or BL22 as a PE toxin control or cycloheximide (Sigma) as a positive control. Finally, cell growth inhibition or viability assays were performed to assess the anti-tumor activity of SS1P.

### Flow cytometry

Cells were incubated with 5 µg/mL of MN (mesothelin mAb; cat. # 200-301-A88; Rockland, Gilbertsville, PA). Binding was detected with goat anti-mouse IgG conjugated with Alexa488 (Sigma-Aldrich). The fluorescence associated with the live cells was measured using FACSCalibur (BD Biosciences).

### Cell growth and viability assays

Cell growth was measured by WST assays. Ten thousand NCI-H226 cells were seeded in each well of a 96-well plate as either monolayer or spheroids, and each well was treated with different concentrations of SS1P or BL22 at 24 hours. Cycloheximide at 10 µg/mL was used as a positive control. The samples were incubated at 37°C, 5% CO_2_ for 72 hours, when monolayer samples had reached approximately 80% confluency. Cell viability was measured using the WST-8 Cell Proliferation Assay Kit (Dojindo, Rockville, MD) [13]. Briefly, monolayer cells were incubated with WST-8 for 2–3 hours while spheroids were incubated overnight at 37°C. The absorbance of the sample at 450 nm was measured with a reference wavelength of 650 nm. Cell growth inhibition was expressed as 50% inhibition of cell viability, which is halfway between the level of viability in the absence of toxin and that in the presence of cycloheximide.

Cell viability was assessed by an ATP measurement assay. Ten thousand NCI-H226 cells were seeded in each well of a 96-well plate as either monolayer or spheroids in RPMI growth media, and each well was treated with different concentrations of SS1P or BL22 at 24 hours. The samples were incubated at 37°C, 5% CO_2_ for 72 hours. Cell viability was measured using the CellTiter-Glo Luminescent Cell Viability Assay Kit (Promega, Madison, WI). Briefly, cells were incubated with CellTiter-Glo Reagent and mixed on an orbital shaker for 2 minutes to induce cell lysis and incubated at room temperature for 10 minutes to stabilize luminescent signal. The absorbance of the sample was measured in terms of relative light units using VICTOR3 Multilabel Counter model 1420 (PerkinElmer Life Sciences, Waltham, MA).

To evaluate the anti-tumor cytotoxicity of SS1P on primary mesothelioma cell lines, 5×10^4^/well of cells were seeded in a 24-well plate. The following immunotoxins were added at various concentrations: SS1P (0, 0.1, 1, 10 and 100 ng/mL), BL22, an immunotoxin against human CD22, as a negative control (0, 0.1, 1, 10 and 100 ng/mL) and HB21, an immunotoxin recognizing the human transferrin receptor, as a positive control at 10 ng/mL. Cells were incubated for 96 hours, then washed twice with PBS; fixed with 10% neutral buffered formalin solution (Sigma) at room temperature for 5 minutes, and crystal violet dye at a concentration of 1mg/mL was added and incubated for 5 minutes at room temperature. The cells were washed, dried and destained in 1% acetic acid. Color intensity was determined by a Versamax microplate reader (Molecular Device, Sunnyvale, California) at a wavelength of 595 nm.

### Confocal laser scanning fluorescence microscopy

Confocal laser scanning fluorescence microscopy imaging (Zeiss LSM 710; Carl Zeiss, Oberkochen, Germany) was performed by placing spheroids in an 8-chambered borosilicate coverglass (Thermo Fisher Scientific, Waltham, MA). SS1P was labeled with Alexa Fluor 488 Protein Labeling Kit (Invitrogen) and incubated with spheroids at 10 µg/mL, and 15 µm thick optical sections were imaged every 15 minutes for 16 hours. Images were taken in both phase contrast and green fluorescence. Fluorescence intensity was measured with ImageJ (NIH, Bethesda, MD).

### SiRNA knockdown

NCI-H226 cells were seeded in a 6-well plate at 1.5×10^5^ cells/well in 2 mL of RPMI growth media and incubated for 24 hours. Cells were then transfected with 400 pmol of E-Cadherin siRNA (cat. #4390828; Ambion, Austin, TX) or Silencer siRNA as a negative control (cat. #4390843; Ambion) in serum-free RPMI media with 1% glutamine mixed with Oligofectamine reagent (cat. #12252-011; Invitrogen). After 36 hours of incubation at 37°C, 5% CO_2_, cells were grown as monolayers or spheroids for 24 hours and then treated with different concentrations of SS1P or BL22. The percentage of E-Cadherin knockdown was evaluated via western blot using 150 µg of cell protein lysate per lane. Cell growth inhibition was measured using a WST-8 assay kit.

### Electron microscopy preparation

A spheroid cell pellet was fixed in 4% formaldehyde/2% glutaraldehyde (Tousimis, Rockville, MD) in 0.1M cacodylate buffer (pH 7.4), then post-fixed in 1% osmium tetroxide (Electron Microscopy Sciences, Ft. Washington, PA) in the same buffer. The pellet was dehydrated in a series of alcohol and propylene oxide and then embedded in epoxy resin for thin-section transmission electron microscopy (TEM) using a Hitachi H7600 TEM (Hitachi, Tokyo, Japan) equipped with a charged couple device camera (AMT, Danvers, MA). To quantitatively evaluate cellular junctions, we examined three representative spheroids. In each spheroid, 50 cells were randomly chosen to calculate the numbers of tight junctions, gap junctions, and desmosomes.

For scanning electron microscopy (SEM) using S3000N (Hitachi) analysis, spheroids were treated in tetramethylsilane solution (Electron Microscopy Sciences) at the end of alcohol dehydration and then allowed to evaporate. Spheroids were lightly coated with platinum palladium for SEM examination.

### Immunohistochemistry

Immunohistochemical stains were performed and interpreted at PhenoPath Laboratories (Seattle, WA) with optimized protocols using a panel of standard markers for the diagnosis of MM: mesothelin, cytokeratin 5/6, calretinin, HBME-1, thrombomodulin, and WT-1.

### Western blot analysis

NCI-H226 cells were allowed to grow for 48–72 hours. After seeding until approximately 60% confluent, 2×10^6^ monolayer cells were centrifuged and collected, washed with 1 mL PBS and resuspended in 100 µl immunoprecipitation assay buffer containing 2% SDS and protease inhibitors (“Complete Mini-EDTA Free” protease inhibitor tablet, Roche, Mannheim, Germany) to solubilize cells or spheroids [13]. Four cycles of freezing at −80° and thawing at 37°C were repeated. Protein lysate was centrifuged at 10,000 rpm for 1 minute, supernatant was collected and protein concentration was measured via Coomassie Plus Protein Assay (Thermo Scientific/Pierce, Rockford, IL). Samples containing 50 µg of cell protein lysate per lane were separated by SDS-PAGE, transferred onto PVDF membranes, and incubated with a primary rabbit antibody. The primary antibodies used include E-Cadherin mAb (cat. #3195; Cell Signaling, Danvers, MA), polyclonal anti-ZO-1 (cat. #61-7300; Invitrogen), polyclonal anti-Connexin 32 (cat. #71-0600; Invitrogen), polyclonal anti-Mcl-1 (cat. #4572; Cell Signaling), Bcl-xL mAb (cat. #2764; Cell Signaling), polyclonal anti-BAX (cat. #06-499; Millipore, Temecula, CA), polyclonal anti-BID (cat. #2002; Cell Signaling), or Bcl-2 mAb (cat. #2870; Cell Signaling). Primary antibodies were detected by secondary goat anti-rabbit antibodies conjugated with horseradish peroxidase (HRP; Invitrogen). HRP-conjugated β-actin mAb (cat. #5125; Cell Signaling) was used as a control. Signals were visualized by an enhanced Luminol-based chemiluminescent western blotting detection kit (GE Healthcare, Piscataway, NJ). Western blots shown are representative images of five individual experiments.

### Statistical analysis

Statistical analysis was performed with Prism (version 5) for Windows (GraphPad Software). Raw data were analyzed by “analysis of variance” with Dunnett's and Newman-Keuls multiple comparison post tests. *p* values<0.05 were considered statistically significant.

## Results

### Establishment of human mesothelioma spheroids

**Figure 1:**
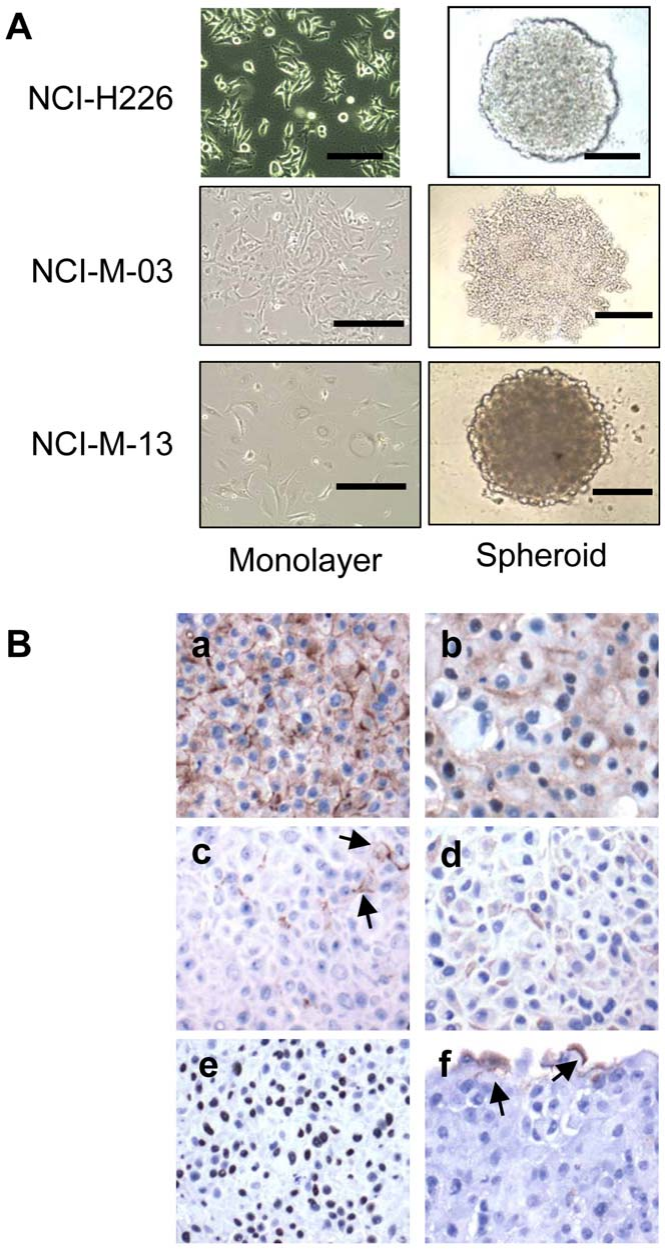
Establishment of human mesothelioma spheroids. **A.** Monolayers and spheroids of human mesothelioma cell line, NCI-H226, and primary mesothelioma lines NCI-M-03 and NCI-M-13. Scale bars, 400 µm (except 200 µm for NCI-M-13 spheroids). **B.** Immunohistochemical staining of an NCI-H226 spheroid (see **Methods**). (a) Mesothelin stains both the membrane and cytoplasm. (b) Calretinin stains both the nuclei and cytoplasm. (c) HBME-1 exhibits a focal membranous staining pattern (arrows). (d) Cytokeratin 5/6 exhibits a focal cytoplasmic staining pattern. (e) WT-1 stains the nuclei. (f) Thrombomodulin exhibits a focal membranous staining pattern close to the rim (arrows).

To investigate the penetration and killing activity of SS1P in mesothelioma microenvironments *in vitro*, we developed 3D tumor spheroids. Spheroids have been observed in the pleural fluid of human MM and linked to increased malignancy [16]. We used the NCI-H226 mesothelioma cell line, which was originally isolated from the pleural fluid of a mesothelioma patient (ATCC), and may also be used to grow clinically relevant MM tumors in mice (MH and MF, unpublished data). We cultured each spheroid from 10,000 cells. After 24 hours of incubation, we found the formation of tight spheroid disks with smooth edges which appeared to be uniform with a diameter of approximately 700 µm with a thickness of 150 µm (Fig. 1A). We also cultured two primary cell lines (NCI-M-03 and NCI-M-13) from MM patients and used the same protocol to make spheroids. As shown in Fig. 1A, NCI-M-13 formed compact mesothelioma spheroids similar to the NCI-H226 spheroids while NCI-M-03 formed mostly aggregates.

To further characterize the NCI-H226 spheroid model, we decided to perform immunohistochemistry analysis with the markers generally used for the diagnosis of MM. Given that there is no overall consensus about how many and which markers should be used, we chose a panel of recommended antibodies for mesothelin, cytokeratin 5/6, calretinin, HBME-1, thrombomodulin, and WT-1 [17]. As shown in Fig. 1B, mesothelin, calretinin and WT-1 were expressed in almost all cancer cells. Interestingly, HBME-1, cytokeratin 5/6 and thrombomodulin were differentially expressed in the spheroid in only a few cancer cells, but not in others. The expression of these markers in spheroids was in general consistent with those of previously published mesothelioma specimens [17], [18].

### Anti-tumor activity on tumor spheroids

**Figure 2:**
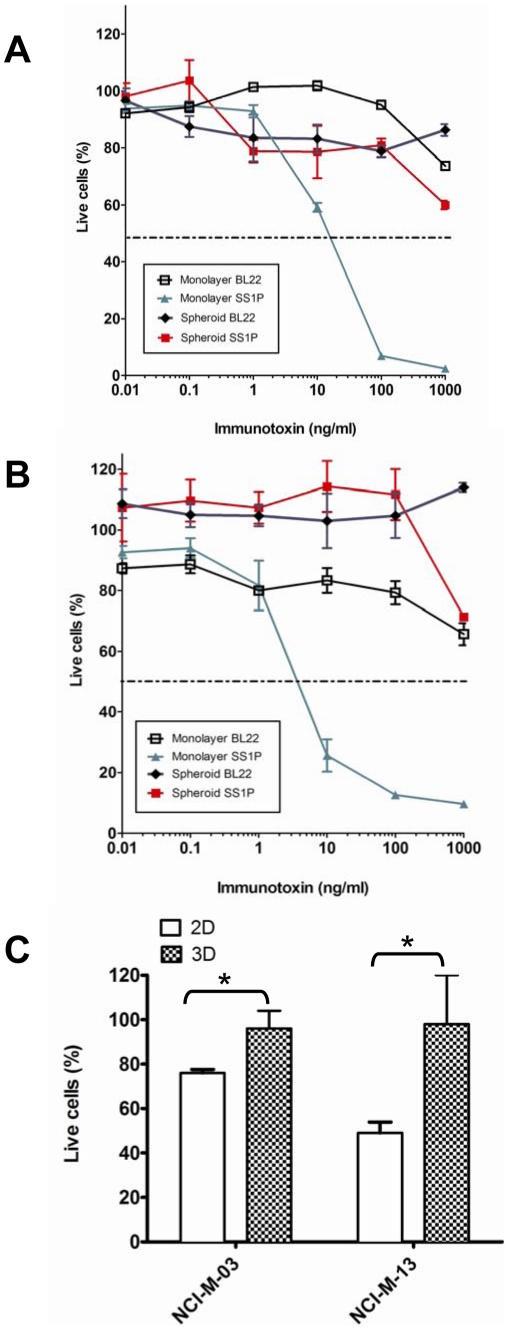
Anti-tumor immunotoxin activity on mesothelioma monolayers and spheroids. Cells treated with SS1P and BL22. **A**. Cell growth inhibition (WST-8 assays) of NCI-H226. IC_50_ of SS1P for spheroids was >1000 ng/mL. IC_50_ for monolayers was ∼10 ng/mL. **B**. Cell viability (CellTiter-Glo Luminescent assays) of NCI-H226. IC_50_ of SS1P for spheroids was >1000 ng/mL. IC_50_ for monolayers was ∼5 ng/mL. **C**. Primary mesothelioma lines NCI-M-03 and NCI-M-13 treated with 100 ng/mL of SS1P. 2D, monolayers; 3D, spheroids. *p<0.01.

We then used SS1P to investigate how tumor microenvironments affect the killing activity and penetration of an antibody agent. The NCI-H226 cells cultured as monolayers and spheroids were treated with SS1P and the anti-CD22 immunotoxin (BL22) was included as a negative control. In cell growth inhibition (WST) assays (Fig. 2A), after 72 hours of immunotoxin treatment, the IC_50_ of SS1P for spheroids was >1000 ng/mL, at least 100 times the IC_50_ for monolayers, ∼10 ng/mL. We confirmed this observation using a cell viability (ATP) assay. As shown in Fig. 2B, the IC_50_ of SS1P for spheroids was >1000 ng/mL and the IC_50_ for monolayers was less than 10 ng/mL. More importantly, in both assays, greater than 50% of the cancer cells from spheroids could not be killed by SS1P concentrations as high as 1,000 ng/mL. Finally, we tested SS1P on primary lines isolated from MM patients. Both lines (NCI-M-03 and NCI-M-13) contain mesothelin-positive tumor cells and mesothelin-negative cells isolated from the same tumor microenvironment. In both cell lines, 100 ng/mL of SS1P killed mesothelioma cells expressing mesothelin, in 20–50% of monolayers (Fig. 2C). However, SS1P was far less effective on spheroids cultured from primary MM cell lines.

### Mesothelin expression in tumor spheroids

**Figure 3:**
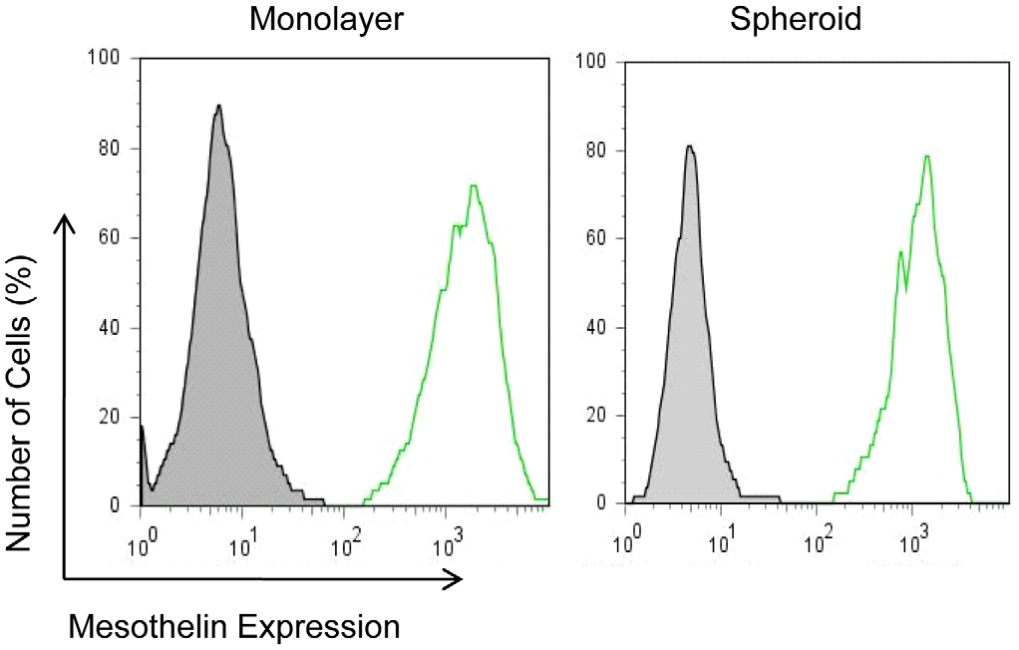
Mesothelin expression in mesothelioma monolayers and spheroids. NCI-H226 cells incubated with an anti-mesothelin mAb (MN) and detected with goat anti-mouse IgG conjugated with Alexa488 by flow cytometry.

Our first step in investigating the possible causes for increased drug resistance was to determine whether or not it was associated with the reduction of antigen expression. This was done by measuring the cell surface expression of mesothelin in NCI-H226 tumor spheroids by flow cytometry. As shown in Figure 3, the mean fluorescence intensity was 1367 for monolayer cells and 1034 for spheroid cells. This showed that the expression of mesothelin between monolayer and spheroid cells is comparable and indicated that the drug resistance of spheroids to SS1P was not due to a reduction of mesothelin expression.

Since SS1P was not able to kill greater than 50% of the NCI-H226 cells cultured as a spheroid at saturated concentrations, yet was able to kill all of the cells cultured as a monolayer (Fig. 2), we hypothesized that such drug resistance may be partially due to the poor penetration of SS1P within tumors.

### Penetration of immunotoxin in tumor spheroids

**Figure 4:**
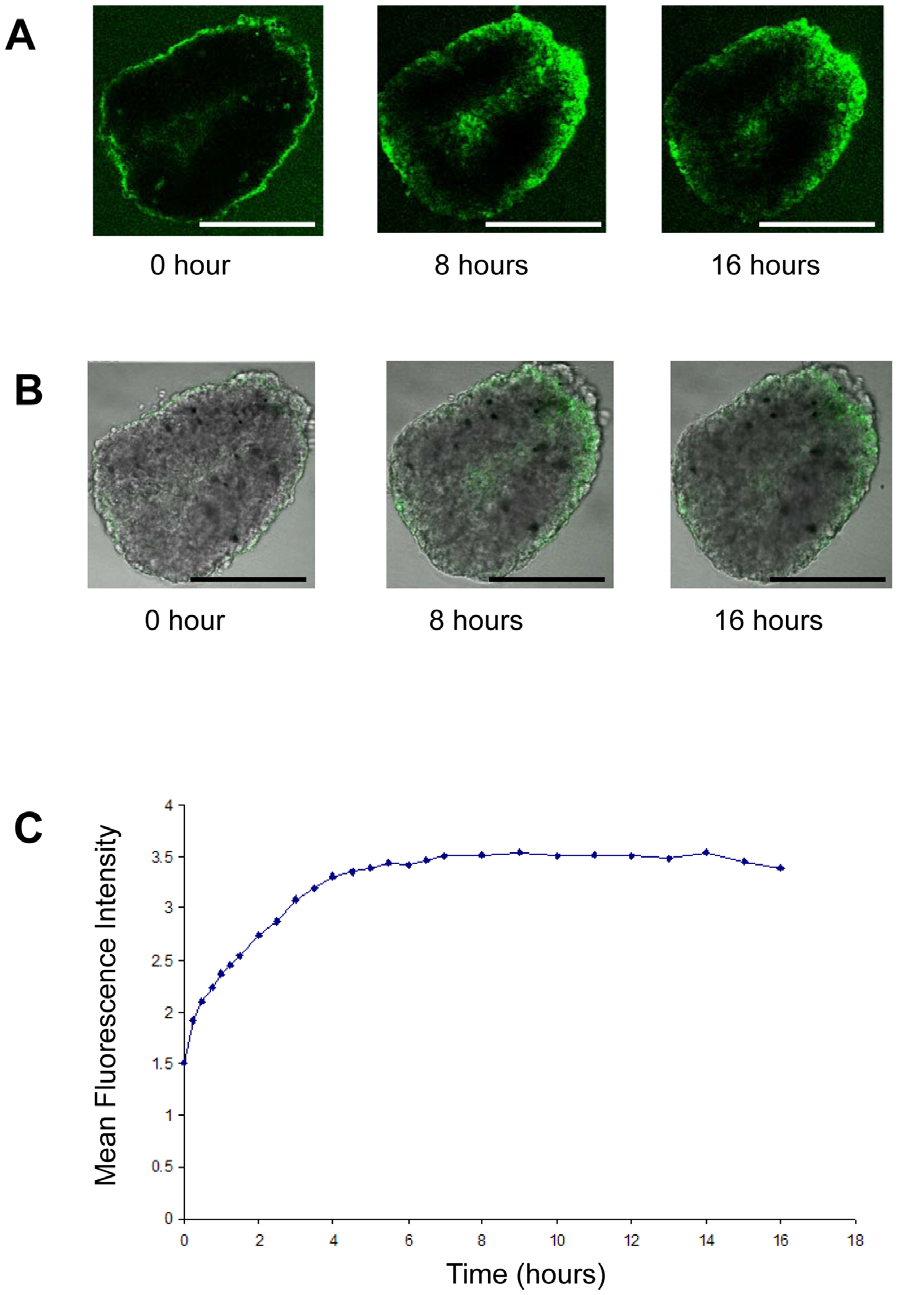
Penetration of Alexa488-labeled immunotoxin SS1P in tumor spheroids. **A**. SS1P labeled with Alexa488 (green fluorescence). A cross section close to the middle of an NCI-H226 spheroid evaluated at hours 0, 8 and 16 using confocal microscopy. **B**. Overlay of bright field and fluorescence images of a spheroid. **C.** Mean fluorescence intensity of SS1P (see **Methods**). Scale bars, 400 µm.

We made a fluorescence-labeled SS1P molecule and developed a strategy to examine the time-lapse penetration of immunotoxin in spheroids by confocal microscopy (supplemental videos S1 and S2). We labeled SS1P with Alexa488 (green fluorescence) and evaluated the cross section close to the middle of an NCI-H226 spheroid at hours 0, 8 and 16 using confocal microscopy (Fig. 4A and 4B). At hour 0, the green fluorescence was confined to the outer surface of the spheroid, and spread towards the center of the spheroid at hour 8 without ever reaching the center. We then quantitatively measured the fluorescence intensity and showed the increase in green fluorescence (SS1P) until 4 hours, after which the intensity plateaued (Fig. 4C). The results demonstrate the incomplete penetration of SS1P.

Given that the mesothelin expression between spheroids and monolayers was similar, even at a saturated concentration of SS1P, an incomplete penetration is unlikely to be caused by the depletion of SS1P. We therefore postulated that the incomplete penetration of the drug may be attributed to a multicellular resistance involving cell contact in spheroids.

### Cell contact in spheroids

**Figure 5:**
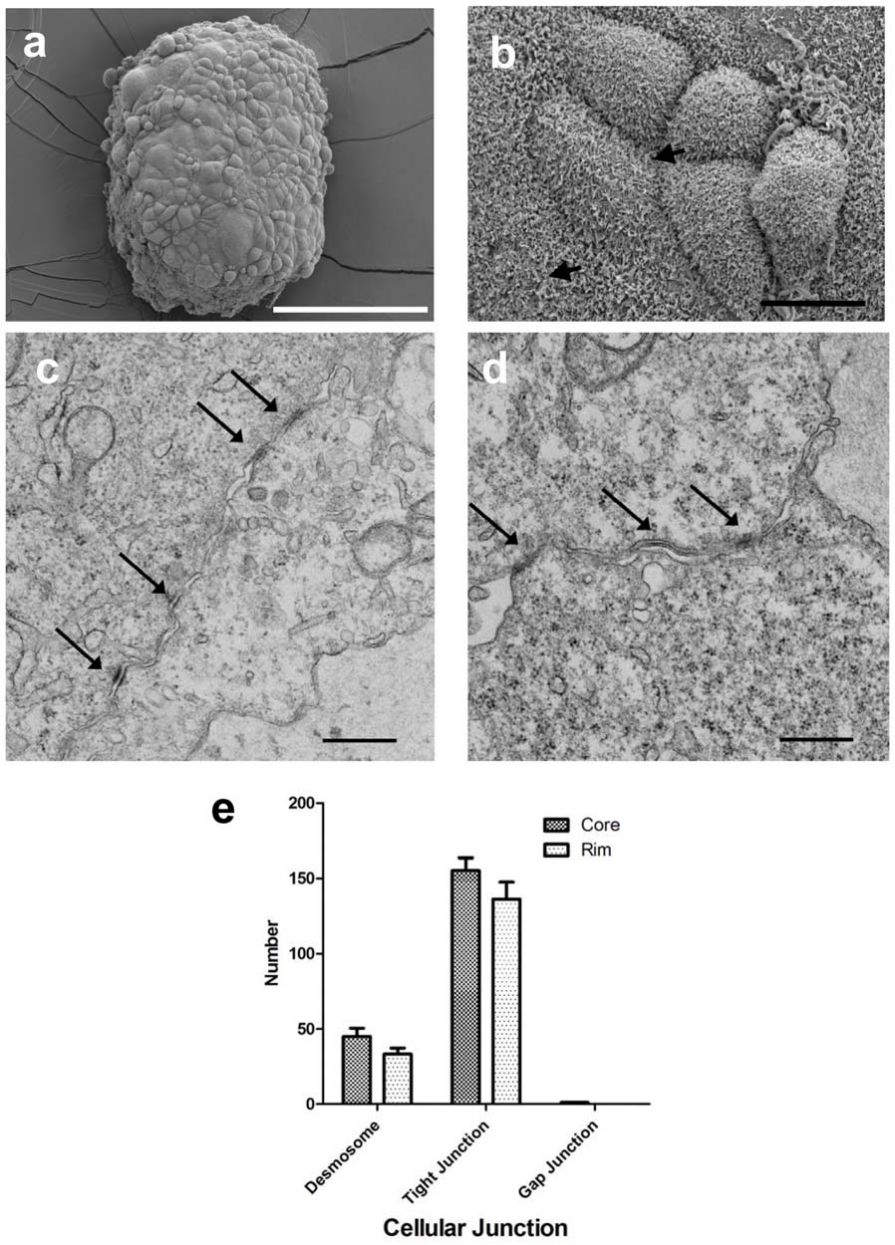
Electron microscopy of cell contacts in NCI-H226 mesothelioma spheroids. **A** and **B**. SEM analysis showing microvilli (arrow). Scale bars, 200 µm (A) and 10 µm (B). **C** and **D**. Ultrathin sections (TEM). Presence of intracellular tight junctions (arrow). Scale bar, 0.5 µm. **E**. Number of cellular junctions in the core and rim areas.

To investigate cellular contact in spheroids, we studied the ultrastructure of spheroids by SEM (Fig. 5A and 5B) and TEM (Fig. 5C and 5D). Interestingly, SEM images (Fig. 5B) show the presence of long and branching microvilli on cell surfaces, a feature characteristic of well-differentiated MM *in vivo* [19].

As shown in Figure 5 (C, D and E), TEM results show that the overall number of tight junctions in spheroids is the highest among cell contacts. Interestingly, the number of tight junctions and desmosomes seems higher in the core area than the rim area in spheroids; however, such an increase is modest based on TEM analysis of three representative spheroids (see **Methods**) (Fig. 5E) (*p*>0.05). Only a few gap junctions are present in spheroids.

The results we obtained from SEM and TEM analysis strongly suggest that mesothelioma spheroids contain characteristic features of MM *in vivo* and that a greater number of tight junctions may contribute to the multicellular resistance that we observed under confocal microscopy in the preceding experiment.

### Expression of cell junction proteins

**Figure 6:**
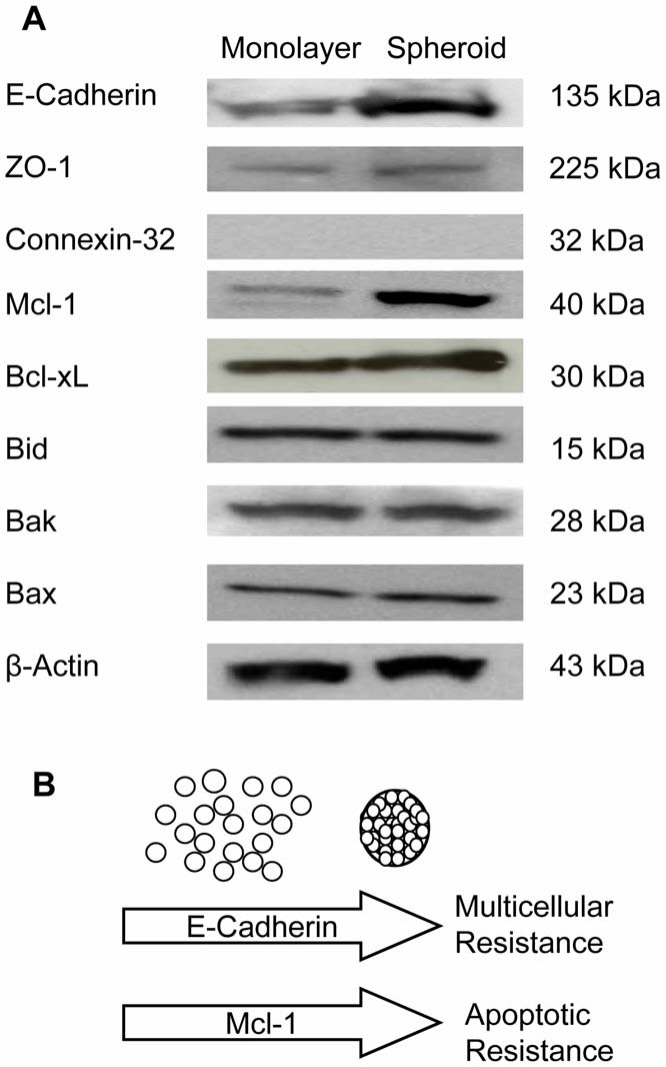
Expression of cell junction and Bcl-2 signaling proteins in NCI-H226 monolayers and spheroids. **A**. Protein expression of E-Cadherin, ZO-1, Connexin-32, Mcl-1, Bcl-xL, Bid, Bak, Bax, and β-actin examined by Western blot. **B**. Molecular mechanisms underlying drug resistance in tumor spheroids.

Based on TEM observations, we hypothesized that an altered expression of intercellular junction molecules might be involved in the poor penetration of SS1P in mesothelioma spheroids. As shown in Figure 6, we examined the expression of a panel of cell contact proteins. Expression of E-Cadherin, a protein involved with tight junctions, was significantly increased in spheroids. ZO-1, another tight junction protein, was also modestly increased in spheroids. However, Connexin-32, the protein responsible for the formation of gap junctions, was absent in both monolayers and spheroids. The elevated expression of E-Cadherin in spheroids found in our study may be related to the increase in the number of tight junctions, since E-Cadherin plays a crucial role in their sealing and assembly [20].

### Bcl-2 signaling proteins

Previous studies on B cells of chronic lymphocytic leukemia showed that the mitochondria-dependent Bcl-2 signaling pathway of apoptosis plays a critical role in the killing of targeted cancer cells by immunotoxins [21]. To examine the potential effects of Bcl-2 signaling on the resistance of SS1P in tumor spheroids, we examined the protein expression of several Bcl-2 signaling molecules in spheroids and monolayers (Fig. 6). Figure 6A shows an increase of prosurvival Mcl-1 in spheroids as compared to monolayers. The expression of Bid, Bak and Bax was not changed in spheroids whereas the expression of Bcl-xL was modestly increased in spheroids. The expression of Bcl-2 was not detectable in either spheroids or monolayers. The increased expression of Mcl-1 may play a role in the inhibition of immunotoxin-induced apoptosis in spheroids. A previous study indicated that high expression of Mcl-1 in 3D lung cancer spheroids based on the H1299 cell line caused its drug resistance [22].

### E-Cadherin siRNA silencing and antibody inhibition

**Figure 7:**
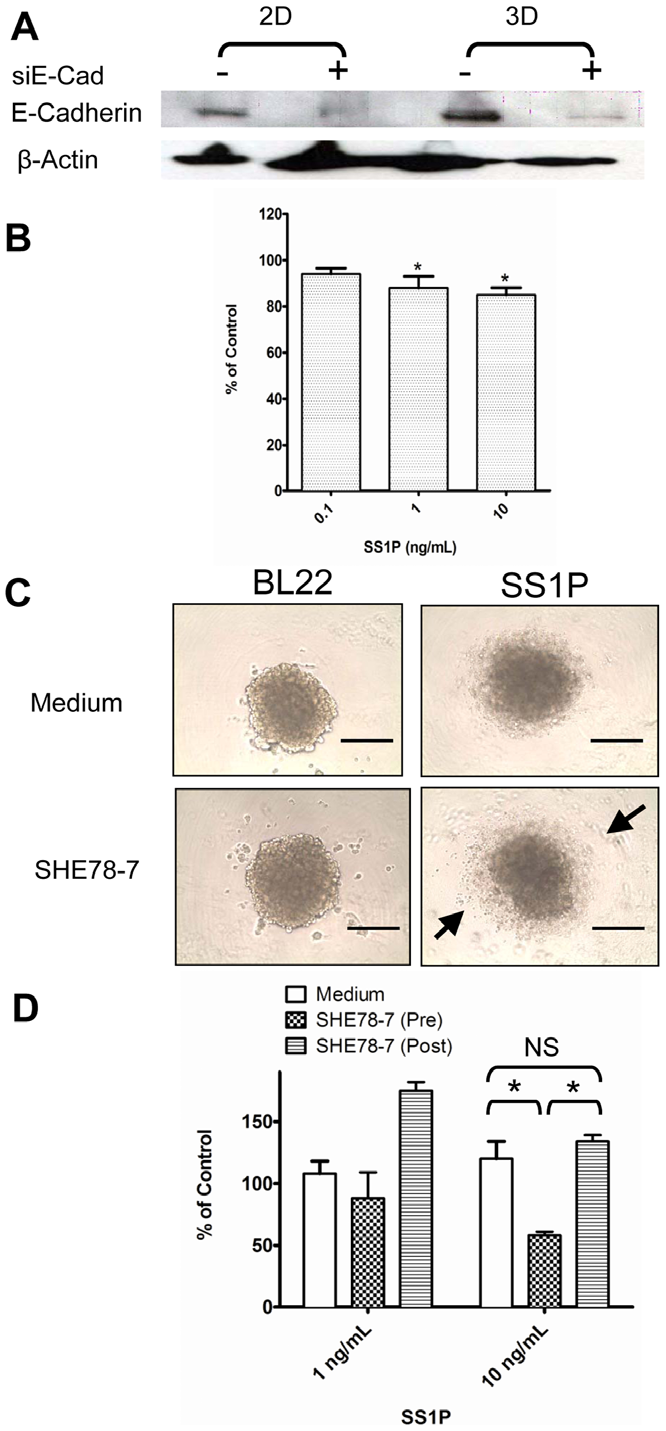
Sensitizing spheroids to immunotoxin therapy *in vitro*. **A**. Silencing E-Cadherin expression by siRNA. siE-Cad, E-Cadherin siRNA. 2D: monolayers; 3D: spheroids. **B.** Silencing E-Cadherin enhanced the anti-tumor activity of SS1P. Cell growth was assessed by incubation with WST-8 with values normalized (%) relative to the growth of the non-silencing siRNA (* *p*<0.05). **C and D**. An anti-adhesive mAb targeting E-Cadherin (SHE78-7) enhanced the anti-tumor activity of SS1P when added before the formation of spheroids (Pre) (* *p*<0.05), but not after (Post). Arrow, lysed cell debris. Scale bars, 400 µm. NS, not significant.

To further investigate the role of E-Cadherin in the penetration of SS1P, we pursued two experimental approaches. First, we silenced the expression of E-Cadherin using siRNA. As shown in Fig. 7A, greater than 80% of E-Cadherin protein expression was reduced in both monolayers and spheroids. As compared to the off-target siRNA control, E-Cadherin-specific siRNA silencing sensitized the cells to immunotoxin therapy at 1 ng/mL and 10 ng/mL of SS1P while E-Cadherin knockdown had little enhancement at 0.1 ng/mL of SS1P (Fig. 7B). Furthermore, we added an inhibitory mAb (SHE78-7) against E-Cadherin and found that it significantly enhanced the anti-tumor activity of SS1P if the mAb was added before the formation of spheroids (Fig. 7C and 7D). However, when SHE78-7 was added after the formation of spheroids, it did not improve SS1P immunotoxin therapy (Fig. 7D).

## Discussion

In the present study, we developed a simple 3D mesothelioma spheroid model to study how tumor microenvironments affect the penetration and killing activity of SS1P *in vitro*. We found that SS1P was not able to kill greater than 50% of mesothelioma cells cultured as a spheroid, but killed all of the cells cultured as a monolayer. To understand this disparity in cytotoxicity, we made fluorescence-labeled SS1P molecules and established an approach based on time-course confocal microscopy to examine the penetration of SS1P within spheroids. We characterized and quantitatively measured the number of cell junctions in spheroids by electron microscopy. In addition, we found that the expression of E-Cadherin, a major protein involved in the sealing and assembly of tight junctions, was significantly increased in spheroids.

Two Phase I clinical trials of SS1P were completed at the NCI in mesothelioma and ovarian cancer patients [12]. Based on Phase I clinical studies showing the safety of SS1P and its demonstrated anti-tumor activity, a clinical trial of SS1P in combination with chemotherapy is currently ongoing in patients with newly diagnosed pleural mesothelioma. The combination of SS1P with chemotherapy is based on results from *in vivo* mouse models showing marked synergy between SS1P and chemotherapy [23]–[26]. Our results indicate that poor penetration is a major mechanistic factor for the resistance of SS1P in mesothelioma. Antibody-based drugs enter into solid tumors mainly by a slow process of diffusion. Due to the fact that immunotoxins have a relatively short life in circulation (20 minutes in mice and 2–8 hours in humans) [27], the time that a tumor is exposed to high immunotoxin concentrations is relatively short. Hence, improving the penetration of immunotoxins is believed to have an important impact on their therapeutic effect. In an initial proof-of-concept study, we pursued two different approaches. In the first approach, we used siRNA to reduce greater than 80% of the protein expression of E-Cadherin and found that the anti-tumor activity of SS1P was significantly enhanced. In the second approach, we used a mAb (SHE78-7) targeting E-Cadherin to block the adhesive function of E-Cadherin [28]. Interestingly, we found that the inhibitory antibody sensitizes the spheroids to immunotoxin therapy if the antibody was added before the formation of spheroids but not after. In the present study, we showed how silencing E-Cadherin expression or blocking its adhesive function does not completely disrupt spheroids, indicating that E-Cadherin is not essential for the formation of mesothelioma spheroids. This observation is consistent with previous studies indicating participation of integrins, not E-Cadherin, in the early stages of spheroid formation [29]. In fact, inhibition of integrins has been shown to enhance breast cancer therapy [30], [31]. Nevertheless, previous studies have also shown that E-Cadherin plays an important role in the morphological transition from loose cell aggregates to compact spheroids [29]. The fact that both siRNA silencing and antibody inhibition targeting E-Cadherin enhanced immunotoxin therapy in the NCI-H226 spheroid model warrants further investigation of E-Cadherin as a target for mesothelioma therapy.

It has been suggested that the Bcl-2 signaling pathway of apoptosis plays an important role in the killing of targeted cancer cells by immunotoxins [21]. A recent study illustrated that the overexpression of Mcl-1 and Bcl-xL inhibited PE immunotoxin-induced cancer cell death [32]. A previous study also revealed that high expression of Mcl-1 in 3D lung cancer spheroids caused its drug resistance [22]. In the present study, we demonstrated an increase of Mcl-1 in spheroids as compared to monolayers, indicating that the 3D mesothelioma spheroid model had acquired Bcl-2 signaling apoptotic resistance as well as multicellular resistance.

This work is one of the first to investigate immunotoxins in 3D tumor spheroids *in vitro*. The method described may allow for further investigations of the tumor microenvironmental effects on drug penetration and tumor cell killing and has applications for the studies of other tumor-targeting antibodies and immunoconjugates *in vitro*. We also understand that some features of solid cancers that are not modeled by spheroids include the influence of stroma and immune cells. Nevertheless, spheroids offer an advantage based on the ability to examine the distribution of drugs in the absence of complicating factors such as pharmacokinetics, which often differ between mice and humans [33].

We believe this method has additional applications for identifying novel molecular targets in tumors. We show in this work that E-Cadherin is highly expressed in 3D mesothelioma but not in monolayers. Interestingly, E-Cadherin is among one of the most important biomarkers that have been proposed as useful in the diagnosis of MM [34], [35]. It will be of great interest to further evaluate E-Cadherin and other cell adhesion molecules as potential therapeutic targets in mesothelioma.

## Supporting Information

Video S1 Time-lapse penetration of immunotoxin in spheroids by confocal microscopy (green fluorescence).
(9.60 MB AVI)

Video S2 Time-lapse penetration of immunotoxin in spheroids by confocal microscopy (overlay of bright field and fluorescence images).
(9.74 MB AVI)
